# A calcium‐mediated actin redistribution at egg activation in *Drosophila*

**DOI:** 10.1002/mrd.23311

**Published:** 2019-12-27

**Authors:** Anna H. York‐Andersen, Qinan Hu, Benjamin W. Wood, Mariana F. Wolfner, Timothy T. Weil

**Affiliations:** ^1^ Department of Zoology University of Cambridge Cambridge UK; ^2^ Department of Molecular Biology and Genetics Cornell University Ithaca New York

**Keywords:** actin organization, calcium wave, *Drosophila* development, Egg activation, oocyte‐to‐embryo transition

## Abstract

Egg activation is the essential process in which mature oocytes gain the competency to proceed into embryonic development. Many events of egg activation are conserved, including an initial rise of intracellular calcium. In some species, such as echinoderms and mammals, changes in the actin cytoskeleton occur around the time of fertilization and egg activation. However, the interplay between calcium and actin during egg activation remains unclear. Here, we use imaging, genetics, pharmacological treatment, and physical manipulation to elucidate the relationship between calcium and actin in living *Drosophila* eggs. We show that, before egg activation, actin is smoothly distributed between ridges in the cortex of the dehydrated mature oocytes. At the onset of egg activation, we observe actin spreading out as the egg swells though the intake of fluid. We show that a relaxed actin cytoskeleton is required for the intracellular rise of calcium to initiate and propagate. Once the swelling is complete and the calcium wave is traversing the egg, it leads to a reorganization of actin in a wavelike manner. After the calcium wave, the actin cytoskeleton has an even distribution of foci at the cortex. Together, our data show that calcium resets the actin cytoskeleton at egg activation, a model that we propose to be likely conserved in other species.

## Graphical abstract

Initiation of egg activation in *Drosophila* results in a dispersal of the actin cytoskeleton, which is required for a calcium wave to pass through the egg and set in motion key downstream events. This calcium wave, in turn, is required for reorganization of the actin cytoskeleton, which occurs in a wavelike manner. This study suggests a highly coregulated calcium and actin signaling network in *Drosophila* oocytes during egg activation.

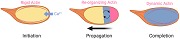

## 1 INTRODUCTION

Production of female gametes is an important part of the faithful passage of genetic information from parents to offspring. Upon completion of oogenesis, mature oocytes remain developmentally arrested. They are triggered to resume development by egg activation, a series of conserved processes that include completion of meiosis to generate a haploid female pronucleus, structural changes in the extracellular matrix of the oocyte, translation and degradation of maternal messenger RNAs (mRNAs), rearrangement of the cytoskeleton, and modification of proteins (Horner & Wolfner, 2008; Kashir, Nomikos, Lai, & Swann, 2014; Krauchunas & Wolfner, 2013; Miao & Williams, 2013; Sartain & Wolfner, 2013; Swann & Lai, 2016). All of these events are preceded by a transient rise in the intracellular level of free calcium (Bernhardt et al., 2018; Kaneuchi et al., 2015; Kubota et al., 1987; Miao et al., 2012; York‐Andersen et al., 2015). This calcium rise, which can result from influx of extracellular calcium and/or the release of calcium from internal stores, leads to dramatic changes in the mature oocyte that prepare it for further development. Many of these changes require the activity of calcium‐dependent kinases and phosphatases (Horner et al., 2006; Mochida & Hunt, 2007; Takeo, Hawley, & Aigaki, 2010; Takeo, Tsuda, Akahori, Matsuo, & Aigaki, 2006), which alter the phosphoproteome of the egg (Guo et al., 2015; Krauchunas, Horner, & Wolfner, 2012; Presler et al., 2017; Roux, Radeke, Goel, Mushegian, & Foltz, 2008; Zhang, Ahmed‐Braimah, Goldberg, & Wolfner, 2019; reviewed in Krauchunas & Wolfner, 2013).

The trigger that initiates egg activation varies across species. In vertebrates and some invertebrates, egg activation requires sperm entry. Significantly, in other invertebrates, egg activation is independent of fertilization and can be initiated by changes in the ionic environment, variation in external pH, or mechanical pressure (reviewed in Horner & Wolfner, 2008).

In *Drosophila melanogaster*, egg activation is triggered by mechanical pressure during ovulation and does not require sperm (Heifetz, Yu, & Wolfner, 2001; Horner & Wolfner, 2008). When a mature oocyte is ovulated and moves from the ovary to the lateral oviduct, it is thought to experience pressure and fluid uptake. This results in its swelling and a transient wave of increased cytoplasmic calcium that initiates from oocyte poles, predominantly the posterior, due to calcium influx through Trpm channels (Horner & Wolfner, 2008; Hu & Wolfner, 2019; Kaneuchi et al., 2015). This calcium influx and wave can be recapitulated ex vivo by submerging dissected mature oocytes in certain hypotonic buffers (Kaneuchi et al., 2015; York‐Andersen et al., 2015). This experimental approach has shown that initiation of the calcium wave requires a functional actin cytoskeleton, as the presence of the actin polymerization inhibitor cytochalasin D results in a loss of the full calcium wave in the majority of oocytes (York‐Andersen et al., 2015).

Changes in the actin cytoskeleton have previously been observed at fertilization and egg activation in multiple systems. In sea urchins, actin polymerizes around the sperm binding site to form a fertilization cone and facilitates the sperm‐egg fusion that initiates egg activation events (Tilney, 1980). In mouse oocytes, actin is required for TRPV3‐mediated calcium influx during parthenogenetic egg activation with strontium chloride, as the actin polymerization inhibitor latrunculin A blocks the calcium rise through TRPV3 activation (Lee, Yoon, Lykke‐Hartmann, Fissore, & Carvacho, 2016). Furthermore, starfish eggs treated with ionomycin to raise the intracellular level of calcium show a perturbed actin cytoskeleton and fail to display the centripetal movement of filamentous actin (F‐actin). Together, these data highlight potential regulatory interactions between the actin cytoskeleton and calcium at egg activation. However, a clear mechanistic understanding and detailed characterization of the interplay between calcium and actin at egg activation is lacking.

Here we use live imaging, pharmacological disruption, mechanical manipulation, and genetics to determine the relationship between calcium and actin at egg activation in *Drosophila*. We show that actin has a broad distribution, filling in ridges that cover the cortex of the mature oocyte. Upon egg activation, we report dispersal of the actin cytoskeleton concurrent with rounding of the cortex as the egg swells. The change in the actin cytoskeleton is required for the calcium wave. The calcium wave, in turn, is required for the reorganization of the actin cytoskeleton, which also proceeds in a wavelike manner. Moreover, we found that cortical actin is dynamic before and after egg activation as well. Regional pressure on mature oocytes can induce a local calcium rise with no increase of the local actin signal. Together, our data suggest that increased cytoplasmic calcium is necessary for actin reorganization. The co‐dependence of the calcium wave and actin reorganization enables the mature oocyte to undergo the dramatic physiological and molecular changes required for the initiation of further development. Since our model links calcium, a ubiquitous secondary messenger, to actin, a key cytoskeletal component, we expect that the interactions established in this study will be conserved in other species.

## 2 RESULTS

### 2.1 Actin reorganizes and is more dynamic during *Drosophila* egg activation

Throughout *Drosophila* oogenesis, actin plays a role in tissue morphogenesis, signaling cascades, localization of mRNAs, and maintaining the integrity of the cortex (Spracklen, Kelpsch, Chen, Spracklen, & Tootle, 2014; Wang & Riechmann, 2007; Weil, Parton, Davis, & Gavis, 2008). To establish the distribution of the actin cytoskeleton before egg activation in the mature oocyte, we used the actin indicator F‐tractin. Generated from the rat actin‐binding inositol 1,4,5‐trisphosphate 3‐kinase A, F‐tractin has been shown to closely correlate with Phalloidin staining and is suggested to be the least invasive actin probe in *Drosophila* follicle development (Schell, Erneux, & Irvine, 2001; Spracklen, Fagan, Lovander, & Tootle, 2014).

**Figure 1:**
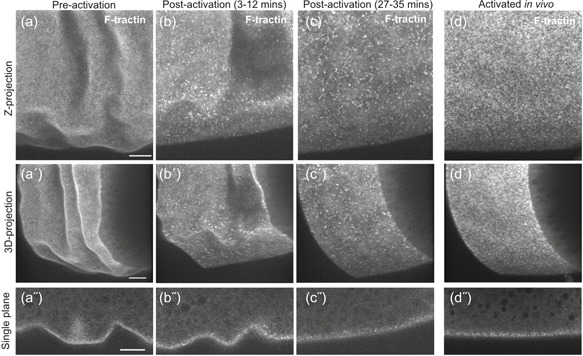
Actin is reorganized at egg activation. Time series of an ex vivo‐activated mature oocyte (a–c; *n* = 6) and in vivo‐activated egg (d; *n* = 4) expressing F‐Tractin.tdTomato. Maximum *Z*‐projection (a–d), three‐dimensional (3D) projection at a 45° angle (a′–d′) and a single *Z*‐plane at 20 µm depth (a″–d″) are shown for each time point. Images were acquired using an Olympus FV3000 confocal microscope. (a–a″) Before the addition of activation buffer, actin is in a smooth cortical distribution around ridges in the cortex (as shown in Movie 1). (b–c″) Following egg activation (3–12 min, as shown in Movie 2) and (27–35 min, as shown in Movie 3), actin is reorganized in larger, more widely distributed foci. 3D projection shows that the cortex has expanded and that actin is no longer associated with the ridge. Single plane images show that a cortical enrichment preactivation is lost and that actin is not as enriched at the cortex after activation. (d–d″) In vivo‐activated eggs collected from the oviduct have a similar actin distribution as mature oocytes activated ex vivo (compare c–c″ [Movie 3] to d–d″ [as shown in Movie 4]). Scale bar = 10 µm (a–d′)

To observe the dynamics of the actin cytoskeleton at live egg activation, we took advantage of an established ex vivo egg activation protocol (Weil et al., 2008) which enabled us to collect high‐resolution three‐dimensional (3D) images at different time points. This showed that before egg activation, actin is finely distributed around well‐defined ridges in the dehydrated mature oocyte (Figure 1a–a″, Movie 1). Following egg activation ex vivo, actin initially dispersed and was then reorganized into larger, more widely distributed foci (Figure 1b–c″, Movies 2 and 3). We observed the same reorganization of actin in the in vivo‐activated egg (Figure 1d–d″, Movie 4) and in the early embryo (Figure S1, Movie 5).

**Figure 2:**
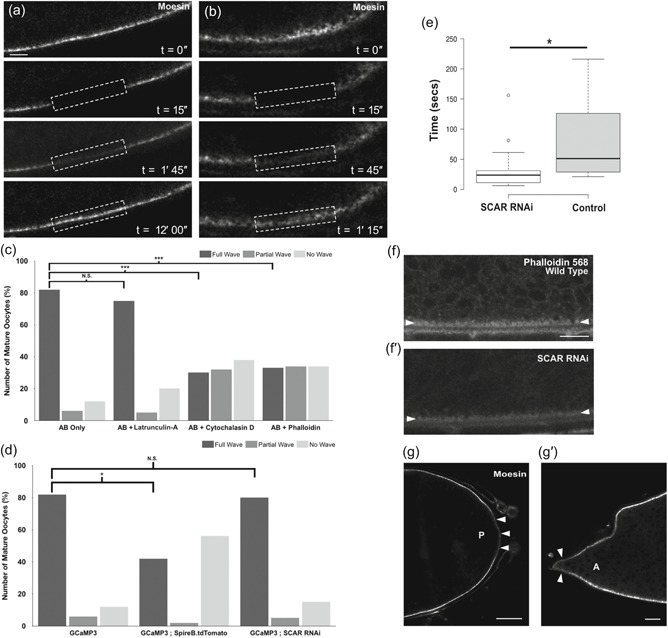
A dynamic actin cytoskeleton is required for a calcium wave at egg activation. (a–b′,g,g′) Egg chamber expressing GFP‐moesin. Images were acquired using an Olympus FV3000 confocal microscope. (a,b) Fluorescence recovery after photobleaching (FRAP) of GFP‐moesin before and after ex vivo egg activation (*n* = 5). GFP‐moesin labeling actin at the lateral cortex is reduced to 25% of original fluorescent intensity following a bleaching step. In both Stage 14 mature egg chambers before activation (a) and after activation (b) recovery can be detected. In the unactivated oocyte, full recovery is complete at 12 min post‐bleaching. However, in the activated oocyte recovery is substantially faster post‐activation (14 vs. 45 s recovery halftime, *n* = 5). Note that movement caused by the addition of activation buffer (AB) meant that recovery could only be recorded for 1 min and 15 s postbleach. The dashed white box denotes the bleached region. (c) Graph showing calcium wave phenotypes when mature oocytes are treated with AB and the actin perturbing drugs latrunculin A, cytochalasin D, or phalloidin. A “full wave” indicates the calcium wave traversed the whole oocyte, a “partial wave” indicates that the calcium wave initiated but did not traverse the whole oocyte, and a “no wave” phenotype indicates that a calcium wave never initiated. When only AB is added, 82% of oocytes displayed full waves. The addition of latrunculin A shows a nonsignificant decrease in full waves to 72% (*n* = 20; *p* = .5). Mature oocytes treated with cytochalasin D or phalloidin show a significant decrease in full waves observed to 28% and 32%, respectively (*n* = 35 for each, Fisher's exact statistical analysis, *p* = .0001 [***]). (d) Graph showing calcium wave phenotypes when mature oocytes are treated with AB in GCaMP3 controls, overexpression of Spire B and knockdown of *scar*. When Spire B is overexpressed there is a significant decrease, to 40%, in the number of activated mature oocytes that show a full calcium wave, and an increase in the number of “no wave” phenotypes to 58% (*n* = 12, Fisher's exact statistical analysis, *p* < .05). When *scar* is knocked down using RNAi, there were no significant changes in the calcium wave phenotype. (e) Boxplot showing the speeds of calcium entry into the mature oocyte in control (GCaMP3) and *scar* RNAi mature oocytes (*n* = 15 and *n* = 18). Speed of calcium entry was determined as the time taken from the addition of AB to the time of first GCaMP fluorescence detection in the oocyte. Initial calcium entry was observed by eye and confirmed through quantification of the fluorescence signal, using a threshold of a 10% increase from the oocyte background. Images were taken every 3 s. There was a significant decrease in the time taken for calcium to enter the oocyte in the *scar* RNAi background (*p* < .05). (f,f′) Stage 14 egg chambers fixed and labeled for actin with phalloidin (*n* = 10). More actin is detected at the cortex in a wild‐type egg chamber (f) compared with a *scar* RNAi expressing egg chamber (f′). White arrowheads mark the lateral cortex of the egg chamber. (g,g′) In the Stage 14 egg chamber, before egg activation, GFP‐moesin is less enriched at the posterior pole (g) of the oocyte (white arrowheads) as compared with elsewhere of the oocyte cortex (paired *t* test *p* < .05, *n* = 20). Observation of GFP‐moesin at the anterior pole was challenging due to the presence of the dorsal appendages. However, in some (~40%; *n* = 25) oocytes Moesin is depleted compared with the lateral cortex (g′) and in these oocytes the depletion is significant (paired *t* test *p* < .05, *n* = 10). Single plane image (a,b,f,g). Scale bar = 10 µm (a,b), 20 µm (f,g)

To test the dynamics of actin before and after egg activation, we performed fluorescence recovery after photobleaching (FRAP) on mature oocytes expressing GFP‐moesin, which is comprised of the C‐terminal actin‐binding domain of moesin, a transmembrane protein, and member of the ERM family, labeling only actin at the cortex (Edwards, Demsky, Montague, Weymouth, & Kiehart, 1997). By measuring the mean fluorescence intensity, we observed a more rapid recovery of GFP‐moesin after photobleaching in activated versus mature eggs. Pre‐activation, GFP‐moesin has recovery halftime of 45 s, compared with post‐activation, where the recovery halftime is 14 s (*n* = 5; Figure 2a,b). We have also tested the recovery of the actin marker Act5C‐GFP (Weil, Forrest & Gavis, 2006), and find similar FRAP recovery times before and after egg activation (41  vs. 20 , respectively [*n* = 5; Figure S2]). The difference in GFP‐moesin (and Act5C‐GFP) recovery before and after egg activation suggests an increased turnover of cortical actin and/or stabilization of F‐actin, either of which could result in increased recruitment of actin markers.

### 2.2 Dynamic actin is required for a calcium wave during egg activation

The dispersal of actin appeared to occur before the previously‐established timing of the calcium wave associated with egg activation (Kaneuchi et al., 2015; York‐Andersen et al., 2015). Moreover, previous work suggested that the calcium wave might depend on a freely dynamic actin cytoskeleton (York‐Andersen et al., 2015). To confirm this relationship, we treated mature oocytes expressing the calcium indicator GCaMP3 with activation buffer (AB) mixed with phalloidin, a class of filament stabilizing phallotoxins (Cooper, 1987). Upon the addition of this solution, the mature oocytes swelled but exhibited a disrupted calcium wave in 68% of the oocytes, as the majority of phenotypes observed were partial or no waves (Figure 2c, *n* = 35). We also elaborated on previous data and showed that when mature oocytes were treated with AB and cytochalasin D, 72% displayed a disrupted calcium wave (Figure 2c, *n* = 35; York‐Andersen et al., 2015). Notably, full calcium waves, defined as a wave of calcium which traverses the entire oocyte, were observed in some oocytes. This is likely due to incomplete inhibition by the drug, and in these cases, swelling of the oocyte occurred as normal. Higher concentrations of the drugs did not change the observed calcium wave phenotypes.

In addition to disrupting F‐actin, we tested the effect of reducing the pool of globular actin (G‐actin) monomers by treating mature oocytes with AB and latrunculin A, which binds the G‐actin monomers and prevents actin assembly (Yarmola, Somasundaram, Boring, Spector, & Bubb, 2000). Upon the addition of this solution, the majority of oocytes showed a full calcium wave (75%; *n* = 20), not significantly different from wild‐type. Together, these pharmacological disruptions suggest that the calcium wave is dependent on a dynamic F‐actin network and can occur without de novo actin assembly from G‐actin monomers (Figure 2c).

We next hypothesized that excess polymerized F‐actin in the mature oocyte would disrupt the calcium wave. To test this, we overexpressed the actin nucleation factor Spire B (Dahlgaard, Raposo, Niccoli, & St Johnston, 2007; Quinlan, Heuser, Kerkhoff, & Dyche Mullins, 2005; Wellington et al., 1999) and observed the calcium wave. We found that additional Spire B leads to a reduction (42% vs. 85%) in the number of mature oocytes that showed a wild‐type calcium wave (Figure 2d; *n* = 12 and *n* = 30). In those eggs that did show a calcium wave, we observed a delay of 5–10 min in the initiation of the wave. These results corroborate the phalloidin data that showed excess or a stabilized actin cytoskeleton to be inhibitory to the calcium wave at egg activation.

Finally, we tested if reducing F‐actin assembly before egg activation would allow for faster entry of calcium into the egg at activation. We used RNAi against *scar*, which encodes an activator of the Arp2/3 complex, to disrupt the nucleation of the actin network (Zallen et al., 2002). We showed that while the number of mature oocytes that showed a normal calcium wave is equal to wild‐type, knockdown of *scar* resulted in calcium entering the egg more quickly than in controls (Figure 2e). Moreover, we observed a decrease in the concentration of actin at the cortex in *scar* knockdown egg chambers (Figure 2f–f′). This supports a model where a spreading or reduction of actin at the cortex allows for calcium entry. In addition, we observed that the cortical actin marker GFP‐moesin was less concentrated at the posterior pole (Figure 2g), and in some cases at the anterior pole (Figure 2g′). This could explain why there is a higher occurrence of calcium waves from the posterior pole of mature oocytes when activated ex vivo.

Taken together, these data support a model in which an intact and dynamic F‐actin cytoskeleton, but not de novo assembly of F‐actin, is essential to regulate the initiation of the calcium wave.

### 2.3 A wavefront of actin follows the calcium wave during egg activation

**Figure 3:**
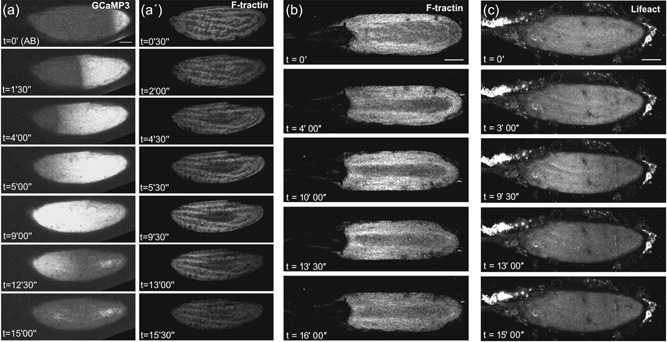
Actin wavefront dynamics follow calcium changes at egg activation. Time series of a mature egg chamber coexpressing GCaMP3 and F‐Tractin.tdTomato (a), or only expressing F‐Tractin.tdTomato (b) or Lifeact::mCherry (c). (a) At activation with activation buffer (AB) (*n* = 9), a calcium wave (a) initiates from the posterior pole (*t* = 0′) and propagates across the entire egg. A corresponding wavefront of F‐tractin (a′) initiates from the posterior pole and traverses the egg. Analysis of the fluorescence intensity shows that the calcium wave initiates on average 106 s after the addition of AB (*SEM* = 11.5 s), traverses the whole oocyte by 267 s (*SEM* = 42 s) and has recovered back to its initial level by 737 s (*SEM* = 148.37 s). A wave of filamentous actin (F‐actin) follows behind this calcium wave, initiating on average 220 s after the addition of AB (*SEM* = 60 s). The actin wave traverses the whole oocyte by 503 s (*SEM* = 72 s) and has recovered to its initial state by 829 s (*SEM* = 222 s). On average, the F‐actin wave lags behind the calcium wave by 103 s. As shown in Movie 6. Images were acquired using an inverted Leica SP5 confocal microscope sequentially, with a scan time of approximately 30 s per *Z*‐stack. (b) F‐actin, labeled by F‐tractin (*n* = 15), shows a posterior to anterior wavefront following the addition of AB (*t* = 0′). The wave initiates (*t* = 4′), propagates across the oocyte to the anterior pole (*t* = 10′) and recovers (*t* = 16′). As shown in Movie 7. Images were acquired using an inverted Leica SP5 confocal microscope. (c) F‐actin, labeled by Lifeact (*n* = 10), shows a similar actin wavefront as F‐tractin (b) in its initiation (*t* = 3′), propagation (*t* = 9′30″) and recovery (*t* = 15′). The bright fluorescence outside of the egg chamber is ovarian tissue associated with the dissection, as shown in movie 6, 7, and 9. Images were acquired using an inverted Leica SP5 confocal microscope. Image a projection of 40 µm (a,b,c). Scale bar = 60 µm (a–c). AB, activation buffer; *SEM*, standard error of the mean

We next examined actin dynamics following the calcium wave in ex vivo egg activation. We used a germline driver to express markers for both calcium and F‐actin simultaneously during *Drosophila* egg activation. Using AB to activate the mature oocyte, we observed and analyzed a calcium wave and a wavefront of actin (Figure 3a–a′, Movie 6). The analysis shows that the actin wavefront (Figure 3b, Movie 7) lags behind the calcium wave at initiation by approximately 2 min and completes propagation across the oocyte approximately 4 min after the calcium wave. A similar phenomenon is observed with the recovery of both waves, actin trailing calcium (see Figure 3a legend for details). We confirmed this result using modified Robb's Buffer (RB, Hu & Wolfner, 2019) to initiate egg activation and show that the speed of the F‐tractin wavefront is similar to that of the calcium wave (0.56 μm/s; Figure S3, Movie 8). The average completion times of the calcium wave and the F‐actin wavefront highlights the similarities between the dynamics of these waves.

To verify the discovery of an actin wavefront at *Drosophila* egg activation, we also tested the widely used in vivo actin marker Lifeact, a 17‐amino acid peptide from yeast Abp140 (Riedl et al., 2008). At egg activation, Lifeact showed an increase in fluorescence from the posterior pole that propagated across the oocyte in a wavelike manner (Figure 3c, Movie 9). The speed of the wave was consistent with that seen with F‐tractin. Overall, using multiple activation methods and F‐actin markers, we demonstrate that the calcium wave during egg activation is followed by a wavefront of F‐actin reorganization.

### 2.4 The calcium wave is necessary to sustain the actin wavefront

**Figure 4:**
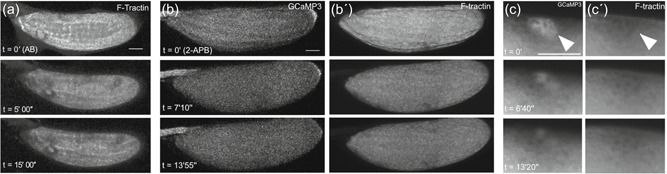
Actin wavefront requires the calcium wave at egg activation. Time series of mature egg chambers expressing F‐Tractin.tdTomato (a) or mature egg chambers coexpressing (b,c) GCaMP3 and F‐Tractin.tdTomato (b′,c′). (a) Time series of mature egg chambers expressing F‐Tractin in a *sarah* mutant background (*sra* ^*A108*^/*sra* ^*A426*^) (*n* = 7). The addition of activation buffer to mature egg chambers causes an initial dispersion of cortical actin (*t* = 4′), but does not initiate a wavefront of filamentous actin (F‐actin) (*t* = 15′). Images were acquired using an inverted Leica SP5 confocal microscope. (b–b′) Addition of 2‐aminoethoxydiphenyl borate (a TRP and IP3 pathway inhibitor) to modified Robb's Buffer prevents initiation of the calcium wave and an F‐actin wavefront is not observed (*t* = 13′55″; *n* = 14). Images were acquired using a Zeiss LSM880 confocal microscope. (c–c′) Application of pressure to the lateral cortex of the mature egg chamber induces a local increase calcium (c) (white arrowhead, *t* = 0′) (*n* = 12). This local increase recovers over time (*t* = 9′30″). Application of pressure does not result in a local F‐actin increase (c′) (white arrowhead, *t* = 0′), nor does it trigger an F‐actin wavefront. Images were acquired using a Zeiss LSM880 confocal microscope. Image a projection of 40 µm (a) or 90 µm (b,c). Scale bar = 60 µm (a–c)

Our previous observation that the actin wavefront follows the calcium wave with similar speed and direction, suggests that the actin wavefront may be dependent on the calcium wave. To test this dependence, we used a genetic approach to assess the requirement of a calcium increase for the F‐actin wavefront at egg activation. Our previous work has shown that the calcium wave does not occur in a *sarah* mutant (York‐Andersen et al., 2015). *sarah* encodes a key protein in the calcium signaling pathway that regulates the calcium‐dependent phosphatase calcineurin (Horner et al., 2006; Takeo et al., 2006, Takeo et al., 2010). F‐actin visualized in a *sarah* mutant background revealed normal swelling of the mature oocyte but no change in the in F‐actin distribution or levels (Figure 4a; *n* = 12).

Next, we inhibited the calcium wave upon egg activation with the addition of 2‐aminoethoxydiphenyl borate (2‐APB). Calcium waves initiate at the oocyte pole(s) due to the influx of external calcium through the TRPM channel (Hu & Wolfner, 2019). Propagation of the waves through the oocyte requires the inositol trisphosphate receptor (IP_3_R, Kaneuchi et al., 2015). 2‐APB is a known ion channel inhibitor that has been experimentally used to block both TRP channels and IP_3_R (Clapham, Runnels, & Strübing, 2001; Maruyama, Kanaji, Nakade, Kanno, & Mikoshiba, 1997). We observed that the addition of 2‐APB results in no calcium wave or F‐actin wavefront, despite the oocytes swelling normally (Figure 4b–b′; *n* = 14), again supporting the conclusion that the calcium wave is necessary for the F‐actin wavefront.

To test the sufficiency of the calcium rise in the induction of an actin wavefront, we initiated a regional calcium rise via local pressure with a microneedle. To ensure that the induced calcium rise was not caused by calcium leaking in due to a damaged plasma membrane (PM), we included propidium iodide (PI) dye in the isolation buffer (IB) to indicate PM damage. We observed that regional pressure by a microneedle can induce a local calcium rise without damaging the oocyte PM (Figure 4c). However, a calcium rise induced by such local pressure did not spread beyond the induction site or change the actin network (Figure 4c′; *n* = 12). This argues that regional pressure can cause a local calcium rise, but that an additional factor(s) is required to reorganize the actin cytoskeletal network.

We therefore assessed whether sustaining a global calcium signal is sufficient to maintain the increase we observed in F‐actin. To test this, we treated mature oocytes with sodium orthovanadate (Na_3_VO_4_), an ATPase inhibitor, dissolved into AB (5a–a′). Upon addition of AB with Na_3_VO_4_, the calcium rise no longer recovered to basal levels and we found that the F‐actin signal also remained elevated. These data suggest that a prolonged global calcium rise may be sufficient to maintain a higher actin signal postactivation and that there is an ATP‐dependent mechanism in recovery and extrusion of calcium after activation. However, we make this suggestion with caution, as Na_3_VO_4_ has numerous effects on cells.

**Figure 5:**
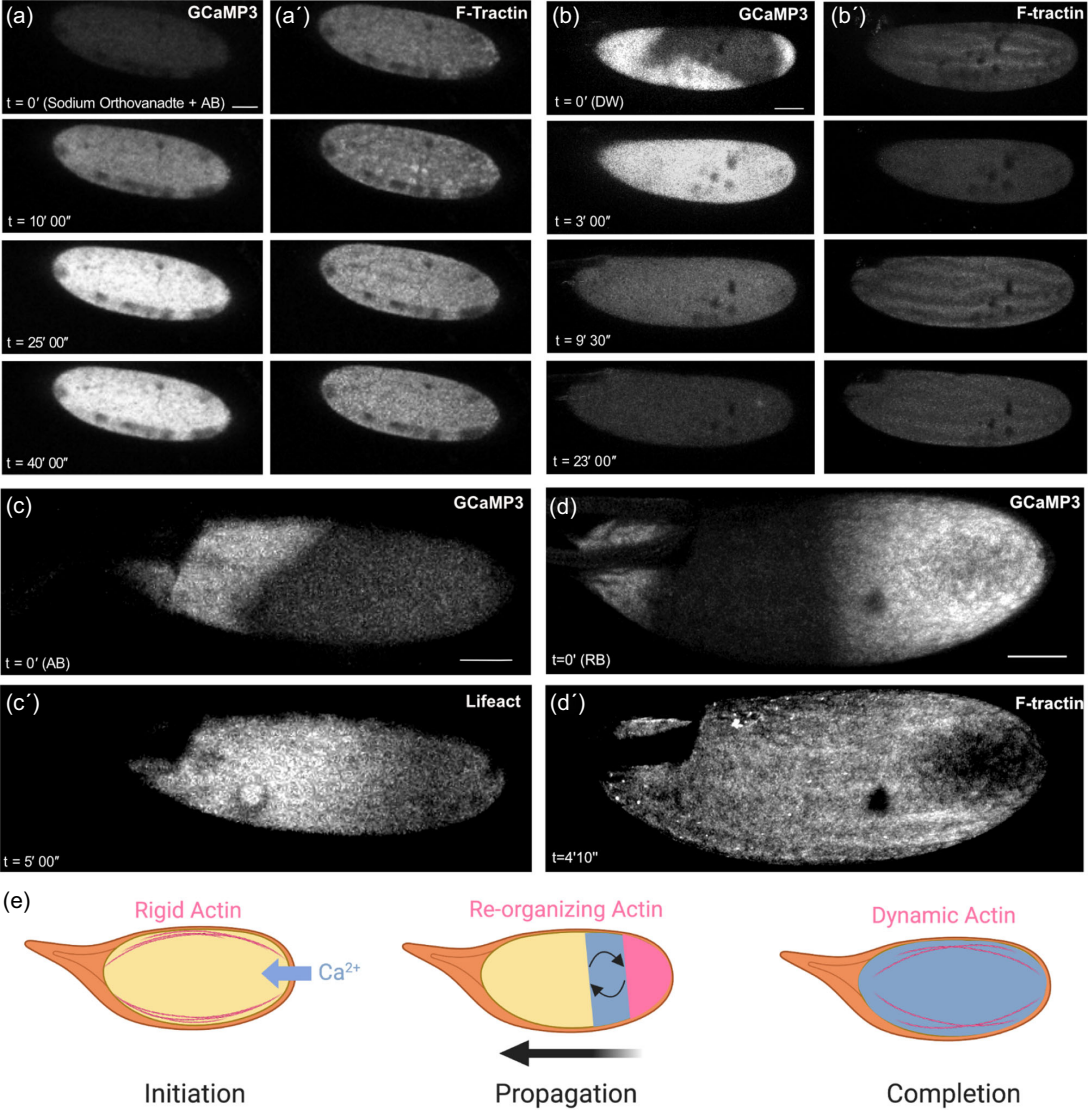
The pattern of actin reorganization follows calcium changes. Time series of mature egg chambers coexpressing GCaMP3 and F‐Tractin.tdTomato (a,b,d) or coexpressing GCaMP3 and Lifeact::mCherry (c). (a–a′) The addition of activation buffer (AB) with sodium orthovanadate initiates a calcium wave that is sustained (*t* = 40′; *n* = 10). An increase in filamentous actin (F‐actin) is similarly seen and is also maintained (*t* = 40′). Images were acquired using an inverted Leica SP5 confocal microscope. (b–b′) Following the addition of distilled water (DW; *n* = 20), the mature egg chamber undergoes a global increase in intracellular calcium (*t* = 0′) which slowly recovers to basal levels (*t* = 15′). F‐actin displays an initial dispersion (*t* = 3′) followed by a global increase (*t* = 9′30″), as shown in Movie 10. Images were acquired using an inverted Leica SP5 confocal microscope. (c) Following the addition of AB (*n* = 5), the calcium wave initiates from the anterior pole (*t* = 0′) and is followed by the wavefront of F‐actin from the same pole (*t* = 5′). Images were acquired using an inverted Leica SP5 confocal microscope. (d) Following addition of modified Robb's Buffer (RB) (*n* = 4), the calcium wave initiates from both anterior and posterior poles (*t* = 0′) and traversing the oocyte (*t* = 4′10″). The F‐actin wavefront initiates at the same sites as the calcium wave (*t* = 4′10″) and propagates with similar characteristics. Images were acquired using a Zeiss LSM880 confocal microscope. (e) Diagram showing the interaction between calcium and actin during *Drosophila* egg activation. Cortical actin is relatively rigid before egg activation. It is less dense near the oocyte poles, where calcium waves initiate ex vivo. The calcium wave that progresses through the oocyte precedes a wavefront of actin reorganization. The actin wave and the calcium wave display similar speeds and are interdependent for propagation and completion. After completion of the calcium wave, cortical actin appears more dynamic in distribution. Actin is represented in pink and calcium in blue. The diagram was created with BioRender.com. Image a projection of 40 µm (a–c) or 90 µm (d). Scale bar = 60 µm (a–d) [Color figure can be viewed at wileyonlinelibrary.com]

To further investigate the dynamics of actin following a calcium rise, we observed the F‐actin phenotype when mature oocytes were treated with distilled water. This treatment results in a rapid swelling of the oocyte and a calcium increase from multiple points at the cortex (5b, Movie 10). Following this pattern of calcium rises, we observed a similar pattern of actin increase approximately 4 min later (5b′). Interestingly, this assay suggests that all regions of the cortex have the potential to initiate a calcium wave that spreads through the oocyte and that stressing the system with excessive fluid uptake results in the loss of spatiotemporal control of the calcium entry and resulting actin reorganization.

Finally, we observe that in some wild‐type ex vivo egg‐activation events, the calcium wave initiated from the anterior pole or from both poles (Kaneuchi et al., 2015). Consistent with our experimental manipulations of the calcium rise and subsequent actin changes, we found that the actin wavefront started from the anterior pole in all of the oocytes that showed the anterior calcium wave, and from both poles when calcium wave initiated from both poles (5c–d′). Taken together, our data suggest that initiation of the calcium wave is necessary to induce a wavefront of F‐actin reorganization, and a global but not regional calcium rise may be sufficient in maintaining the actin wave and coordinating its directionality.

## 3 DISCUSSION

Actin plays an essential role in fertilization and egg activation in many organisms. However, its functions in these aspects of *Drosophila* reproduction have not been fully explored. In this study, we showed that F‐actin disperses and become more dynamic during *Drosophila* egg activation and that this dynamic actin cytoskeletal network is required for a normal calcium wave to occur during ex vivo egg activation (5e). Moreover, the calcium wave mediates a reorganization of F‐actin in a wavelike manner. This F‐actin wavefront follows, requires, and has similar characteristics to the calcium wave. Together, these findings suggest a highly co‐regulated calcium and actin signaling network in *Drosophila* oocytes during egg activation.

### 3.1 The importance of the actin cytoskeleton in egg activation

Our work demonstrates that in *Drosophila*, actin dynamics are important for the calcium rise which in turn leads to a reorganization of F‐actin. Rearrangement of F‐actin appears to be a common feature of oocytes undergoing egg activation. The reorganization of F‐actin following exposure to osmotic pressure in zebrafish oocytes mediates the release of the cortical granules (Becker & Hart, 1999; Hart & Collins, 1991). More recent work in zebrafish has shown that the actin‐binding factor, Aura, mediates this reorganization of actin and that in a Aura mutant background F‐actin does not rearrange, and cortical granule exocytosis is inhibited (Eno, Solanki, & Pelegri, 2016).

Our data are reminiscent of events in starfish, where a pharmacologically‐induced calcium rise results in depolymerization of surface actin and polymerization of F‐actin bundles within the cytoplasm (Vasilev et al., 2012). Starfish oocytes also exhibit actin rearrangements at egg activation that are required for the calcium release at the cortex (Kyozuka et al., 2008). This calcium wave initiates a PIP_2_ increase at the starfish cortex in a biphasic manner, whereas pharmacological inhibition of PIP_2_ results in a delayed calcium wave and disrupted actin organization (Chun et al., 2010).

### 3.2 Functions of the actin wavefront

It is hypothesized that F‐actin waves are essential for actin self‐reassembly after its initial dispersion at the cortex and are thought to mediate the polymerization of actin (Bretschneider et al., 2009; Case & Waterman, 2011). For example, pharmacological depolymerization of actin results in an increased number of actin waves in *Dictyostelium* (Bretschneider et al., 2009). A similar observation was made in fibroblasts, where actin waves take form of “circular dorsal ruffles,” which are nonadhesive actin structures found on the dorsal side of some migrating cells (Bernitt, Döbereiner, Gov, & Yochelis, 2017; Bernitt, Koh, Gov, & Döbereiner, 2015; Chhabra & Higgs, 2007). Moreover, the F‐actin wave is proposed to be associated with actin‐binding factors, including Arp2/3, myosin B, CARMIL, and coronin. Live visualization of these factors in *Dictyostelium* cells have shown their enrichment at the leading edge of the F‐actin wavefront (Bretschneider et al., 2009; Khamviwath, Hu, & Othmer, 2013). Together, this provides other examples of how the reorganization of F‐actin can trigger F‐actin waves. By analogy, the F‐actin wavefront we observed during *Drosophila* egg activation might serve a similar purpose of reorganizing the actin cytoskeleton to facilitate further developmental events in embryogenesis.

### 3.3 Calcium and actin coregulatory networks

Our work suggests a pathway of actin‐calcium interdependence as initial calcium entry is enabled at the poles, where we see a breakdown of the actin cytoskeleton at the cortex, and this calcium increase is required to generate a global reorganization in the actin population. Calcium is thought to regulate the actin cytoskeleton predominantly via actin‐binding factors, including myosin, profilin, and villin/gelsolin (reviewed in Hepler, 2016). Early experiments showed that calcium regulates muscle contractions via myosin V protein, rather than directly through the actin filaments (Szent‐Györgyi, 1975). In this case, calcium binds troponin that in turn binds tropomyosin to mediate the actin‐myosin contraction cycle (Lehman, Craig, & Vibert, 1994). Similarly, plants utilize the actin‐myosin network for cytoplasmic streaming, and actin is again regulated by calcium signaling via myosin XI (Tominaga et al., 2012). Calcium can also control actin dynamics via profilin, an actin‐binding factor, which is required for F‐actin polymerization (Vidali & Hepler, 2001). Experiments with profilin show that calcium inhibits F‐actin polymerization by sequestering actin monomers and profilin subunits, which are no longer able to form the actin filaments (Kovar, 2000). Calcium causes depolymerization or severing of the actin cytoskeleton via villin actin‐binding factor and was shown to aid the organization of actin in epithelial intestinal cells (Walsh, Weber, Davis, Bonder, & Mooseker, 1984).

One especially interesting factor is α‐actinin, an actin cross‐linking protein that is a member of the spectrin family and is found primarily on F‐actin filaments in non‐muscle cells. α‐actinin has been suggested to cross‐link actin under low calcium conditions. However, when intracellular calcium rises (as in the case of a calcium wave), α‐actinin releases actin filaments, thus no longer cross‐linking the F‐actin filaments (Jayadev, Kuk, Low, & Murata‐Hori, 2014; Prebil et al., 2016; Sjöblom, Salmazo, & Djinović‐Carugo, 2008). However, further work will be required to establish the exact connections between calcium and actin networks (Veksler & Gov, 2009).

### 3.4 An actin and calcium feedback loop during egg activation

One hypothesis for how actin dynamics are linked to calcium wave initiation is through interaction with mechanosensitive channels such as TRP channels and DEG/ENaC. We recently showed that the *Drosophila* TRPM channel is required for the initial calcium influx (Hu & Wolfner, 2019), but how TRPM responds to mechanical stimuli remains to be elucidated. There are several models for how these channels are activated. One suggested cue is direct mechanical stress applied on the PM, due to physical pressure during ovulation and/or osmotic swelling of the oocyte in the oviducts. While it is possible that the TRPM channel might respond to the stress directly, it is also possible that the mechanical signal is also transduced through the actin cytoskeleton causing the channel to open, enabling an influx of calcium ions (Christensen & Corey, 2007).

We showed here that before egg activation, the cortical actin marker Moesin is less concentrated at the posterior end of the oocyte where calcium waves generally initiate and decrease in concentration at the cortex. It is possible that lack of actin rigidity is linked to calcium channel opening: The initial sparse site of actin allows calcium channels to open and leads to calcium influx, which in turn regulates the dynamic changes in actin to form a wavefront following calcium wave. The interlinked nature of these two pathways suggests a positive feedback loop that facilitates the progression and completion of the waves. This could be achieved as follows: (a) actin disperses and calcium enters the oocyte; (b) this calcium rise causes further actin dispersion through regulation of actin‐binding proteins; (c) more calcium then enters the activating egg which results in the complete calcium wave and actin reorganization in a wavelike manner. Overall, we propose that in *Drosophila* egg activation, actin is able to modulate the intracellular calcium rise, and calcium waves in turn regulate the reorganization of actin.

## 4 MATERIALS AND METHODS

### 4.1 Fly stocks and reagents

The following fly stocks were used: *matα4‐GAL‐VP16>UASp‐GCaMP3* (as previously described Kaneuchi et al., 2015); *UAS‐Lifeact::mCherry* (*a* gift from from Palacios Lab, QMUL); *UAS‐Spire B.tdTomato* (*a* gift from the Quinlan Lab, UCLA); *UASp‐F‐Tractin.tdTomato* (RRID:BDSC_58988, 58989); *UASp‐Act5C‐GFP* (RRID:BDSC_7309); *UAS‐SCAR.RNAi* (RRID:BDSC_36121). All UAS constructs were driven with matα4‐GAL‐VP16 (RRID:BDSC_7062, 7063).

Fly stocks were raised on either standard cornmeal–agar medium or yeast‐glucose agar medium at 21°C or 25°C, mainly on a 12‐light/dark cycle. Before dissection of the mature oocytes, female flies were fattened on yeast for 48–72 hr at 25°C.

BAPTA (Sigma‐Aldrich) was used at 10 mM, cytochalasin D (Sigma‐Aldrich) at 10 µg/ml, Phalloidin (Sigma‐Aldrich) at 3.3 nM, latrunculin A (Sigma‐Aldrich) at 10 µg/ml, 2‐APB (Sigma‐Aldrich) at 200 µM, and RFP/mCherry booster (Atto 594) at 1:200 (ChromoTek).

### 4.2 Oocyte and embryo collection

Mature oocytes were dissected from the ovaries from fattened flies using a probe and fine forceps as described previously (Kaneuchi et al., 2015; York‐Andersen et al., 2015). Embryos were collected for 30 min on apple or grape juice agar plates with yeast and then dechorionated in 50% bleach.

### 4.3 Ex vivo egg activation assay

Oocytes were activated with different, but equivalent preparation methods:

1) Dissected oocytes were placed in series 95 halocarbon oil (KMZ Chemicals) on 22 × 40 coverslips, aligned parallel to each other to maximize the acquisition area for imaging and left to settle for 10–15 min before addition of AB and imaging (Derrick, York‐Andersen, & Weil, 2016); AB contains 3.3 mM NaH_2_PO4, 16.6 mM KH2PO4, 10 mM NaCl, 50 mM KCl, 5% polyethylene glycol 8000, 2 mM CaCl_2_, brought to pH 6.4 with a 1:5 ratio of NaOH:KOH (Mahowald, Goralski, & Caulton, 1983).
2) Oocytes were dissected in IB (Page & Orr‐Weaver, 1997) in a glass‐bottomed Petri dish and activated by replacing IB with modified RB. IB contains 55 mM NaOAc, 40 mM KOAc, 1.2 mM MgCl_2_, 1 mM CaCl_2_, 110 mM sucrose, 100 mM HEPES in ddH_2_O. IB is adjusted to pH 7.4 with NaOH and filter sterilized. RB contains 55 mM NaOAc, 8 mM KOAc, 20 mM sucrose, 0.5 mM MgCl_2_, 2 mM CaCl_2_, 20 mM HEPES in ddH_2_O. RB was adjusted to pH 6.4 with NaOH and filter sterilized (Hu & Wolfner, 2019);
3) For the high‐resolution 3D live imaging oocytes were mounted in a glass‐bottomed culture dish (MatTek) in Schneider's insect culture medium (GIBCO‐BRL) with a 1 mm^2^ coverslip on the oocyte. For activation, Schneider's medium was removed and replaced with AB (Weil et al., 2008).

### 4.4 Imaging

Mature oocytes were imaged using a Zeiss LSM880 confocal microscope with Zen software under 10 × 0.45NA water immersion objective with a detection wavelength of 493–556 nm for GCaMP signal, and 566–691 nm for F‐tractin signal. Z‐stacks were taken from the shallowest to deepest visible plane of the oocyte. Z‐stacks and time series of images taken were 3D reconstructed in Imaris software for final output. Image processing and analysis were performed using ImageJ (Schindelin et al., 2012).

Alternatively, images were acquired with an inverted Leica SP5 confocal microscope, under 20 × 0.7NA immersion objective with acquisition parameters of 500–570 nm, 400 Hz. Similar settings were used for the F‐tractin cytoskeleton, with acquiring parameters of 570–700 nm. The Z‐stacks were taken from the shallowest visible plane of the oocyte and were acquired at 2 μm per frame 40‐μm deep. The Z‐stacks were presented as maximum projections of 40 μm, unless stated otherwise.

High‐resolution 3D images of the cortex were collected using an Olympus FV3000 confocal microscope with Schneider's mounting and activation preparation. Parameters for image collection were: 60x silicon immersion objective, 60 μm Z‐stack, 0.34 μm between each Z‐slice, 2,048 × 2,048 resolution of varying ROIs at the cortex, approximately 8–10 min per complete acquisition. Movies 1–5 show the brightest point 3D projection with a *Y*‐axis of rotation and 0.34 µm slice spacing (120 Z‐frames). The display has an initial angle of 0°, total rotation of 150° and a rotation angle increment of 1°. Image processing and analysis were performed using ImageJ (Schindelin et al., 2012) and displayed as a maximum Z‐projection or 3D projections.

FRAP was carried out on the cortex of the mature oocyte using a UV laser on the Olympus FV3000 microscope for 15 s. The fluorescence recovery was recorded using Olympus FV3000 for 20 min. Samples were imaged under a 30 × 1.05NA silicon immersion objective. Despite technical challenges due to swelling of activated eggs, we were able to visualize the bleached region clearly for approximately 100 s after photobleaching. A partial calcium wave is defined as one that initiates but fails to completely traverse the entire oocyte; A full (or complete) calcium wave is defined as one that is able to traverse the entire oocyte.

### 4.5 Microneedle manipulation

Microneedles were fabricated from borosilicate glass rods (Catalog no. BR‐100‐15; Sutter Instrument), in a Sutter model P97 flaming/brown micropipette puller, with the following program: heat = 490, pull = 0, velocity = 25, time = 250. Mature oocytes of the indicated genotype were incubated in a glass‐bottom Petri dish in 100 µl of IB that contained PI (Molecular probes) at a final concentration of 1 µg/ml as an indicator of PM integrity. Since the PM is not permeable to PI, PI staining at the microneedle pressing site would suggest damage to the PM and false calcium signal by calcium in IB entering through PM breach. To manipulate the oocyte, the microneedle was attached to and manipulated with an Eppendorf Injectman NI 2 micromanipulator. The indicated regions of mature oocytes were pressed with the microneedle until a calcium rise occurred as visible with GCaMP. Oocyte calcium and actin dynamics were observed for 20 min.

## CONFLICT OF INTERESTS

The authors declare that there are no conflict of interests.

## Supporting information

Supporting information

Supporting information

Supporting information

Supporting information

Supporting information

Supporting information

Supporting information

Supporting information

Supporting information

Supporting information

Supporting information
