# Supplementary material for: A calcium‐mediated actin redistribution at egg activation in Drosophila
Source: Mol Reprod Dev. 2019 Dec 27;87(2):293–304. doi: 10.1002/mrd.23311 (PMC7044060; doi:10.1002/mrd.23311)
Supplement: Supplementary file 1 — Supporting information [file MRD-87-293-s001.pdf]

SUPPLEMENTAL FIGURE 1

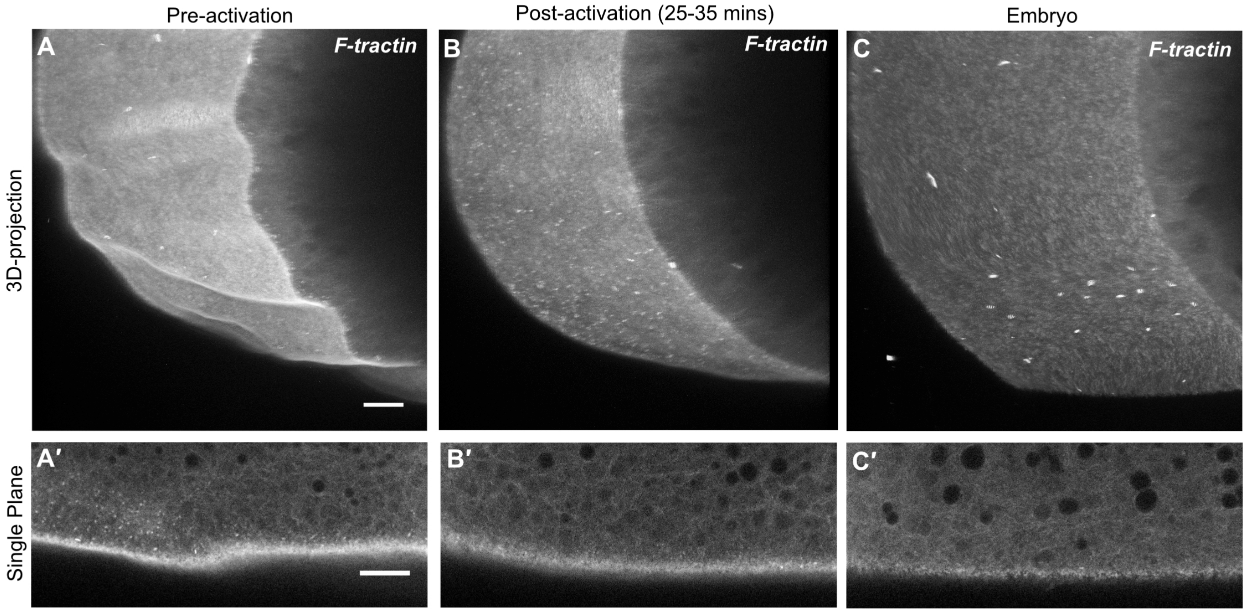

FIGURE S1 Actin is reorganized at egg activation and in the early embryo.

Time series of a mature oocyte activated *ex vivo* with activation buffer and an early embryo (0-30min) expressing F-Tractin.tdTomato. 3D-projection at a 60° angle (A-C) and a single Z-plane at 12μm depth (A′-C′) are shown. Images were acquired using an Olympus FV3000 confocal microscope.

(A-B′) Consistent with Figure 1, actin is distributed around ridges in small foci prior to egg activation and is reorganized into larger more widely distributed foci after egg activation is complete (n=6).

(C-C′) The early embryo (Movie 5, n=5) displays a similar actin distribution and the *ex vivo* and *in vivo* activated eggs (Figure 1D and Figure S1B).

Scale bar, 10 μm (A-C′).

SUPPLEMENTAL FIGURE 2

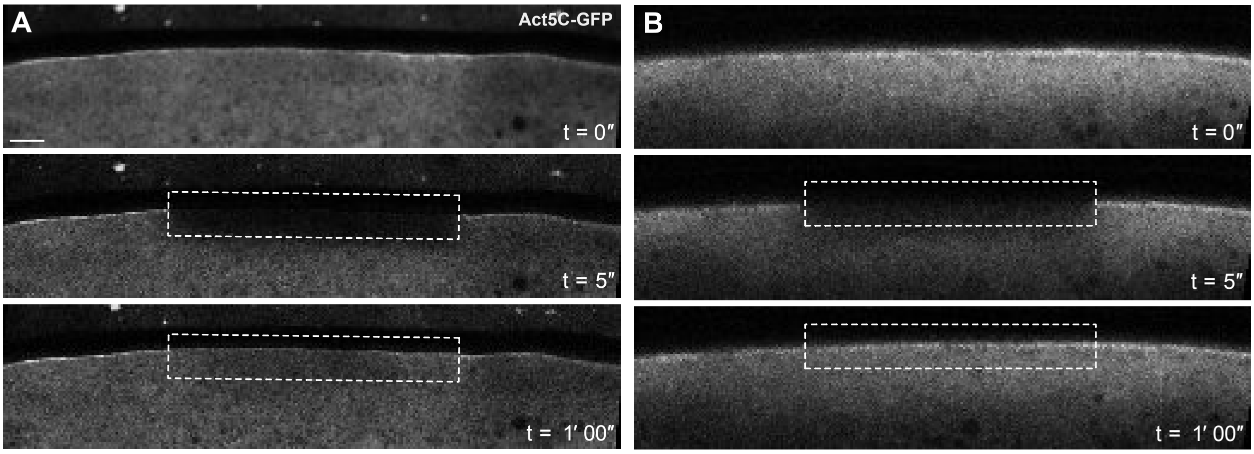

FIGURE S2 Act5C-GFP recovers more quickly after photobleaching post-egg activation.

(A-B) Egg chamber expressing Act5C-GFP Fluorescence recovery after photobleaching (FRAP) before and after *ex vivo* egg activation (n=5). Act5C-GFP at the lateral cortex is reduced to 10% of original fluorescent intensity following a bleach step. In both stage 14 mature egg chambers before activation (A) and after activation (B) recovery can be detected. In the unactivated oocyte, full recovery is complete at 5 minutes post-bleaching. However, in the activated oocyte recovery is substantially faster post-activation (20 seconds versus 41 seconds recovery halftime, n=5). Dashed white box denotes bleached region. Images were acquired using an Olympus FV3000 confocal microscope.

Single plane image (A-B). Scale bar, 10μm.

SUPPLEMENTAL FIGURE 3

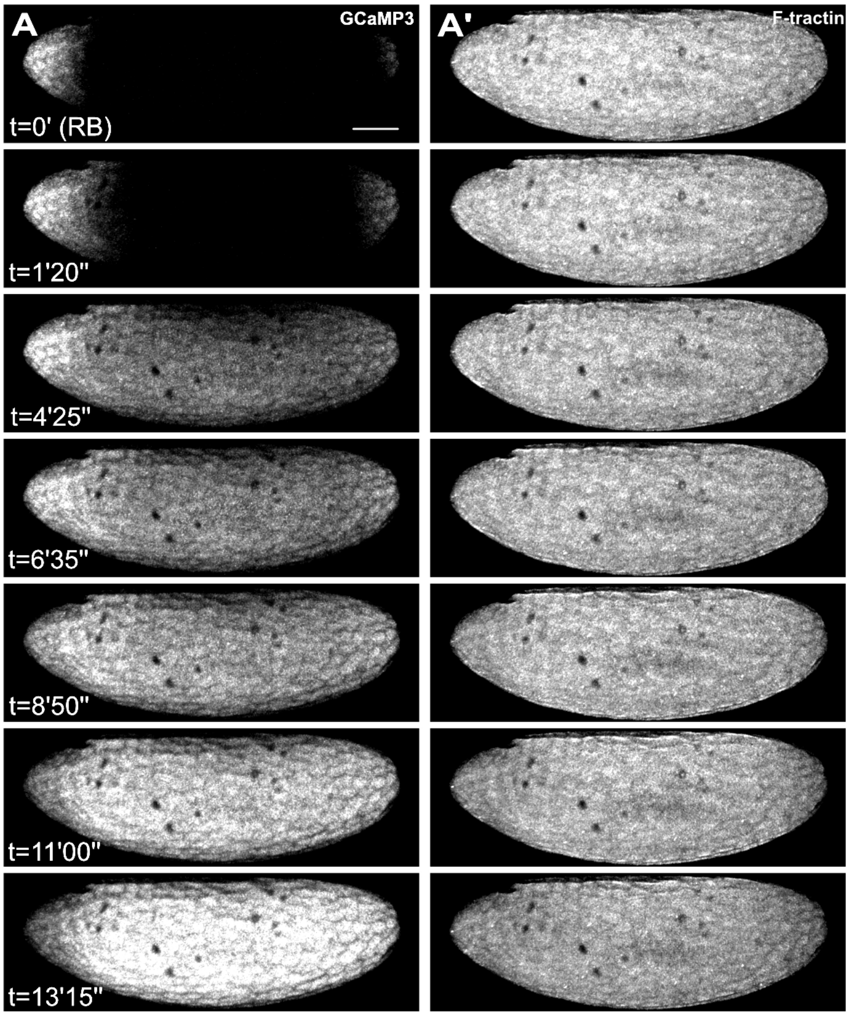

FIGURE S3 A wavefront of actin follows the calcium wave during egg activation

Time series of a mature egg chamber co-expressing GCaMP3 and F-Tractin.tdTomato.

(A-A′) At activation with Robb's buffer (RB) (n=4), a calcium wave (A) initiates from anterior and posterior poles and propagates across the entisre egg. A corresponding wavefront of F-tractin (A′) initiates from both poles and traverses the egg. As shown in Movie 8. Images were acquired using a Zeiss LSM880 confocal microscope.

SUPPLEMENTAL MOVIE LEGENDS:

MOVIE 1. 3D-projection of actin in the mature oocyte before *ex vivo* egg activation.

The data is the same experiment as displayed in Figure 1A′. 3D-projection displayed from 0° to 150° of the lateral cortex of a mature oocyte, before egg activation, expressing F-Tractin.tdTomato. In addition to the cortical actin distribution visible around ridges in the cortex, filaments are also visible emanating away from the cortex. Images were acquired using an Olympus FV3000 confocal microscope.

MOVIE 2. 3D-projection of actin in the mature oocyte during *ex vivo* egg activation.

The data is the same experiment as displayed in Figure 1B′. 3D-projection displayed from 0° to 150° of the lateral cortex of a mature oocyte expressing F-Tractin.tdTomato 3-12 minutes after the addition of activation buffer which induced *ex vivo* egg activation. Actin begins to reorganize as ridges in the cortex are no longer present due to swelling of the egg. Images were acquired using an Olympus FV3000 confocal microscope.

MOVIE 3. 3D-projection of actin in the mature oocyte after *ex vivo* egg activation.

The data is the same experiment as displayed in Figure 1C′. 3D-projection displayed from 0° to 150° of the lateral cortex of a mature oocyte expressing F-Tractin.tdTomato after *ex vivo* egg activation is completed. Following egg activation, actin has a new distribution which is characterized by large foci more widely placed throughout the cortex. Images were acquired using an Olympus FV3000 confocal microscope.

MOVIE 4. 3D-projection of actin in the *in vivo* activated egg.

The data is the same experiment as displayed in Figure 1D′. 3D-projection displayed from 0° to 150° of the lateral cortex of an *in vivo* activated egg expressing F-Tractin.tdTomato. The same actin distribution as in the *ex vivo* activated egg (Movie 3) is observed. Images were acquired using an Olympus FV3000 confocal microscope.

MOVIE 5. 3D-projection of actin in the early embryo.

The data is the same experiment as displayed in Figure S1C. 3D-projection displayed from 0° to 150° of the lateral cortex of an early embryo expressing F-Tractin.tdTomato. The same actin distribution as in the *ex vivo* and *in vivo* activated egg (Movie 3 and 4) is observed. Images were acquired using an Olympus FV3000 confocal microscope.

MOVIE 6. F-actin wave follows the calcium wave at *Drosophila* egg activation with activation buffer.

The data is the same experiment as displayed in Figure 3A-A′. A mature egg chamber co-expressing GCaMP3 (top) and F-Tractin.tdTomato (bottom). Following *ex vivo* egg activation with AB, a calcium wave initiates and propagates from the posterior pole. These events are followed by a reorganization of actin in a wavefront manner. Movies are displayed equivalently and show 16 minutes of developmental time in 2.6 seconds. Images were acquired using an inverted Leica SP5 confocal microscope.

MOVIE 7. F-actin wave labeled with F-tractin at *Drosophila* egg activation.

The data is the same experiment as displayed in Figure 3B. A mature egg chamber expressing F-Tractin.tdTomato shows a wavefront of actin initiating from the posterior pole following *ex vivo* egg activation with AB. Movie shows 20 minutes of developmental time in 4 seconds. Images were acquired using an inverted Leica SP5 confocal microscope.

MOVIE 8. F-actin wave follows the calcium wave at *Drosophila* egg activation in Robb's Buffer.

The data is the same experiment as displayed in Supplemental Figure 3. A mature egg chamber co-expressing GCaMP3 (top, cyan) and F-Tractin.tdTomato (bottom, magenta). Following *ex vivo* egg activation with RB, a calcium wave initiates and propagates from both poles. These events are followed by a reorganization of actin, also from both poles, in a wavefront manner. Movies are displayed equivalently and show 25 minutes of developmental time in 5 seconds. Images were acquired using a Zeiss LSM880 confocal microscope.

MOVIE 9. F-actin wave labeled with Lifeact at *Drosophila* egg activation.

The data is the same experiment as displayed in Figure 3C. A mature egg chamber expressing Lifeact::mCherry shows a wavefront of actin initiating from the posterior pole following *ex vivo* egg activation with AB. Movie shows 18 minutes of developmental time in 4 seconds. Images were acquired using an inverted Leica SP5 confocal microscope.

MOVIE 10. F-actin has similar dynamics to the calcium wave in oocytes treated with distilled water.

The data is the same experiment as displayed in Figure 5B. Mature egg chambers co-expressing GCaMP3 (top) and F-Tractin.tdTomato (bottom). Addition of distilled water results in a calcium increase from multiple points at the cortex which is followed by a similar reorganization of actin. Movies are displayed equivalently and show 24 minutes of developmental time in 2.8 seconds. Images were acquired using an inverted Leica SP5 confocal microscope.
